# Projected health and economic effects of the increase in childhood obesity during the COVID-19 pandemic in England: The potential cost of inaction

**DOI:** 10.1371/journal.pone.0296013

**Published:** 2024-01-24

**Authors:** Iván Ochoa-Moreno, Ravita Taheem, Kathryn Woods-Townsend, Debbie Chase, Keith M. Godfrey, Neena Modi, Mark Hanson

**Affiliations:** 1 School of Human Development and Health, University of Southampton, Southampton, United Kingdom; 2 Centre for Health Economics, University of York, Heslington, United Kingdom; 3 NIHR Southampton Biomedical Research Centre, University Hospital Southampton NHS Foundation Trust, University of Southampton, Southampton, United Kingdom; 4 Southampton City Council, Civic Centre, Southampton, United Kingdom; 5 Southampton Education School, University of Southampton, Southampton, United Kingdom; 6 MRC Lifecourse Epidemiology Centre and NIHR Southampton Biomedical Research Centre, University Hospital Southampton NHS Foundation Trust, University of Southampton, Southampton, United Kingdom; 7 Section of Neonatal Medicine, School of Public Health, Faculty of Medicine, Imperial College London, London, United Kingdom; 8 Partnership for Maternal, Newborn and Child Health, WHO, Geneva, Switzerland; University of Leicester, UNITED KINGDOM

## Abstract

**Background:**

The prevalence of overweight and obesity in young children rose sharply during the COVID-19 pandemic. Here we estimate the potential future health and economic effects of these trends in England.

**Methods:**

Using publicly available annual Body Mass Index (BMI) data from 2006–2022, we calculated the increase in overweight/obesity prevalence (BMI ≥85^th^ reference percentile) during the COVID-19 pandemic among children aged 4–5 and 10–11, and variation by deprivation and ethnicity. We projected the impact of child BMI trends on adult health measures to estimate added lifelong medical and social costs.

**Results:**

During 2020–2021 there were steep increases in overweight and obesity prevalence in children. By 2022, overweight and obesity prevalence in children aged 4–5 returned to expected levels based on pre-pandemic trends. However, overweight and obesity prevalence in children aged 10–11 persisted and was 4 percentage points (p<0.001) higher than expected, representing almost 56,000 additional children. The increase was twice as high in the most compared with the least deprived areas. The additional lifelong healthcare cost in this cohort will amount to £800 million with a cost to society of £8.7 billion. We did not find an increase in maternal obesity associated with the COVID-19 pandemic, however, prevalence grew faster in the post pandemic period.

**Discussion:**

The return of overweight and obesity prevalence to pre-pandemic trends in children aged 4–5 provides a clear policy target for effective intervention to tackle this growing and serious population health concern.

## Introduction

The rapidly rising prevalence of overweight and obesity is a major public health problem globally. The World Health Organisation (WHO) estimates that obesity prevalence has tripled worldwide in the last 40 years [1]. In 2020, around 800 million adults were obese, accounting for 15% of the world’s population, with this number projected to increase to 1 billion by 2030 [2, 3]. Among children and adolescents aged to 5 to 19 years, the prevalence of obesity globally was 8% (157 million) in 2020 and is predicted to reach 19% (250 million) by 2030 [2, 3]. There were 38 million children under 5 years old living with obesity in 2019. However, overweight and obesity do not affect populations evenly, and prevalence is higher among less advantaged groups [4]. BMI also shows large disparities across ethnic groups and the impact of socioeconomic status on obesity varies across ethnicities [5].

Overweight and obesity prevalence rose sharply during the COVID-19 pandemic in many populations in England, with the largest single year increase for decades in younger age groups [6]. The greater effect on weight gain in children than adults may have been caused by the higher physical activity needs of children, the deterioration of healthy eating habits during the period in which the majority of children were schooled from home, the cancellation of organised sports and recreational activities reducing physical activity, and effects on children’s sleeping schedules and screen time [7, 8]. Childhood obesity leads to increased likelihood of obesity in adult life [9], and a concomitant increase in the likelihood of developing multiple long-term conditions including type 2 diabetes, hypertension, and coronary artery disease [10]. These result in a decline in healthy life expectancy, premature mortality and impose high personal, healthcare, societal and financial burdens. Patients living with obesity access healthcare more frequently, require more specialised care, have more admissions to hospital and undergo more surgical procedures [11]. There are also wider societal implications of obesity and its consequences. Childhood obesity is associated with stigma, discrimination, behavioural problems, lower self-esteem, and lower academic performance [12].

A modelling study estimated that the global cost of obesity would be US$3 trillion in 2030 and US$18 trillion by 2060 [13]. In the UK, the NHS spent £6·1 billion on obesity-related ill-health in 2014–2015, while the overall impact on productivity from life years and healthy life years lost was estimated to be £27 billion [14]. In a study conducted before the COVID-19 pandemic, NHS costs attributable to overweight and obesity were projected to reach £9·7 billion by 2050, and the wider annual costs to society estimated to reach £49·9 billion [15]. Treatment interventions for established obesity have had little success in stemming the increase in prevalence, with pharmacotherapy being the primary option for most patients [16]. While new obesity drugs have shown efficacy, the potential side effects and economic and ethical considerations argue for a whole systems-based approach to prevention [17].

To contribute to making the health, economic and social justice case for addressing this challenge, we provide for England a projection of the longer-term health and wider social costs of the increase in the prevalence of overweight and obesity in young children in two age groups during the pandemic, with particular emphasis on this increase in deprived areas and specific ethnic groups.

## Methods

### Data sources

We used data on childhood BMI from the National Childhood Measurement Programme (NCMP) from England. This includes data on children in their first year of formal schooling (reception year, age 4–5) and their last year of primary education (year 6, age 10–11). BMI data were available annually from the school year 2006/2007 to 2021/2022. The data include ethnicity and deprivation category (only until 2021) at national and regional levels. BMI classification is derived by calculating the child’s centile with respect to the British 1990 reference population [6]. The thresholds used are underweight (BMI less than or equal to the 2^nd^ centile), healthy weight (BMI above the 2^nd^ and below the 85^th^ centiles), overweight (BMI greater or equal to the 85^th^ but less than the 95^th^ centile), obese (BMI greater or equal than the 95^th^ centile), and severely obese (BMI greater than or equal to the 99·6^th^ centile) [6]. Therefore, the definition of “obesity” includes “severe obesity”. Deprivation categories are based on the Index of Multiple Deprivation (IMD). This is a combined measure of deprivation based on 39 indicators grouped into 7 domains that reflect an individual’s experience of living in a certain Lower-layer Super Output Area [18]. BMI data were classified by deprivation decile of the school’s postal code as home address postal code is not available.

We estimated BMI persistence into adulthood using data from two longitudinal cohorts from the UK. The National Child Development Study (NCDS) follows a cohort of 17,415 people born in England, Scotland, and Wales in a single week of 1958. The study collects comprehensive information over 12 waves, including BMI data at 7, 11, 16, 23, 32, 42 and 50 years of age [19]. We complemented lifelong BMI data using the English Longitudinal Study of Ageing (ELSA) which includes longitudinal socio-demographic data for a representative sample of the English population aged 50 to 100 years old (y) at recruitment [20].

### Increase in overweight and obesity prevalence

We estimated the increase in overweight and obesity prevalence on the post-pandemic period compared to the pre-pandemic using data from 2006 to 2022. We fitted the prevalence trend for each BMI category by age group as a function of year employing a quadratic polynomial. We explored heterogeneous changes in BMI categories prevalence fitting additional models including sex, deprivation level and ethnicity.

### Lifelong BMI evolution

To measure the persistence of childhood overweight and obesity into adult life, we projected two populations to age 80y, one using pre-COVID-19 prevalence levels as the baseline, and a second using post COVID-19 levels to compare lifelong overweight and obesity prevalence differences. The lifelong BMI categories prevalence projections were made using a two-pronged approach: for ages 11 to 50, we used the NCDS birth cohort; and the ELSA cohort for ages 50 to 80 [19, 20]. We reweighted the NCDS cohort to resemble the pre- and post-pandemic populations at age 11y. The method consisted in assigning different weights to each individual in the NCDS cohort so the BMI-category prevalences were equal to our two populations: one with pre-pandemic BMI levels and the other with post-pandemic BMI levels. Then, for each reweighted population, we predicted BMI-categories prevalence from 11 to 50 years old by modelling an individual’s probability of belonging to a given BMI class across time using multinomial logistic regression. The difference in BMI between the two populations at age 50 would be the due to the differences in BMI-category prevalence at age 11y. For each *k* of the BMI categories *K = 4*, (normal weight, overweight, obese, and severely obese) there is a prevalence *p*_*k*_*(x)* for individuals of age *x*.

We can run 3 binomial logistic regressions for each category *k≠1* prevalence, leaving normal weight, *k = 1* as a pivot, and regressing the other three categories *k = 2*,*3*,*4* against the pivot outcome:

$$ln\frac{p_{k\neq 1}(x)}{p_{k=1}(x)}=\beta_{k}x_{k}$$

We can solve for *p*_*k*_*(x)* to find each prevalence as:

$$p_{k}(x)=\frac{e^{\beta_{k}x_{k}}}{1+\sum_{k=2}^{K}e^{\beta_{k}x_{k}}}$$

Forecast of BMI categories prevalence for 50 to 80 years old are projected using the ELSA cohort. The classes modelled were normal weight (≥18.5 kg/m^2^, <25kg/m^2^), overweight (≥25 kg/m^2^, <30 kg/m^2^), obese (≥30 kg/m^2^, <40 kg/m^2^), and severely obese (≥40 kg/m^2^) [21].

The post-pandemic BMI sample group was also reweighted to have a BMI 1 kg/m^2^ (0.9–1.09) higher than the pre-pandemic group. The difference in BMI chosen was based on a meta-analysis which reported an increase of 2.67 kg and BMI of 0.94 kg/m^2^ in children during lockdowns [22]. To analyse the sensitivity of this parameter, we also calculated the costs for a difference of 0.75 kg/m^2^ and 1.25 kg/m^2^ between the two groups.

### Lifelong costs associated to elevated BMI

We estimated the lifelong cost associated to the COVID-19 pandemic as the difference between the lifelong cost of two predicted populations, one with post-pandemic overweight and obesity prevalence as the baseline, and one with pre-pandemic prevalence. Drawing from the literature risks of morbidity and mortality, we calculate healthcare costs, and the wider social costs.

We estimated the cost of the main health conditions resulting from elevated BMI, namely type 2 diabetes, coronary heart disease, stroke, osteoarthritis and five types of cancer (colorectal, renal, breast, endometrial and oesophageal) [23–26]. We estimated the prevalence of each health condition in our simulated cohorts and multiplied it by the annual healthcare cost per case to obtain the cost per BMI category. We estimated prevalence by applying the risk of developing a condition associated with high BMI compared to normal weight in the population (see S1 Table in S1 File). We obtained annual costs from the literature and inflated these to 2021 £UK (see S2 and S3 Tables in S1 File). Prevalence for the general population and risk factors for the BMI categories were also derived from the literature [27].

### Quality of life

We estimated the impact in terms of losses in utility measured in quality adjusted life years (QALYs). The EQ-5D scores for the UK obtained from the literature (see S4 Table in S1 File) [28, 29]. The impact of obesity and overweight on quality of life was obtained by multiplying the prevalence of each related disease by the utility loss associated with that disease. Annual utility losses across different morbidities were added and multiplied by the cost of a QALY. According to HM Treasury, the monetary value of a loss or gain of a QALY in a wider social perspective is £70,000 in 2020/2021 prices [30]. The National Institute for Health and Clinical Excellence recommends using a monetary value for a QALY of £20,000 - £30,000 for cost-effectiveness analyses, to reflect the opportunity costs of allocating resources elsewhere in the health system [31]. Other studies empirically estimate an even lower value to reflect a substantial opportunity cost of healthcare displacement [32]. For the current study, we considered the willingness to pay for a QALY from a broader social perspective [30].

### Productivity losses

We estimate losses in productivity associated with elevated BMI using a human capital approach. Overweight and obese workers are respectively 1.2 and 1.6 times more likely to take sickness absence days than healthy weight workers [33]. The average days lost due to sickness per worker, according to the Office for National Statistics, was 4.1 in England in 2019 (see S5 Table in S1 File) [34]. Using these data, we predicted the number of productive days lost in our population of children from the 10-11yr age group assuming a labour participation rate of 76% as reported by the Office for National Statistics [34]. We multiplied this by the reported daily mean salary of £89.03 for 2021 and applied it to individuals aged 20 to 65 [34]. Using the average labour participation rate for all population cancels out heterogeneities across sexes and ages.

Productivity losses can occur also due to premature mortality i.e., if individuals die before age 65 which we assume to be the retirement age. We estimated productive years lost due to premature mortality using mortality rates [35] (S6 Table in S1 File) associated with elevated BMI from the literature multiplied by the average annual salary £32,049 for 2021 [34] (see S7 Table in S1 File).

### Discounting

We express all costs in 2021 £UK and provide both undiscounted and discounted estimates. The discount rates follow UK HM Treasury recommendations, with healthcare and productivity costs discounted at 3.5% from year 1 to year 30; 3% from year 31 to 75; and 2.5% after year 75. Utilities are discounted at 1.5% from year 1 to 30; 1.29% from year 31 to year 75; and 1.07% after year 75 (S8 Table in S1 File) [31].

## Results

### Overall effect of the pandemic on childhood BMI

**Fig 1:**
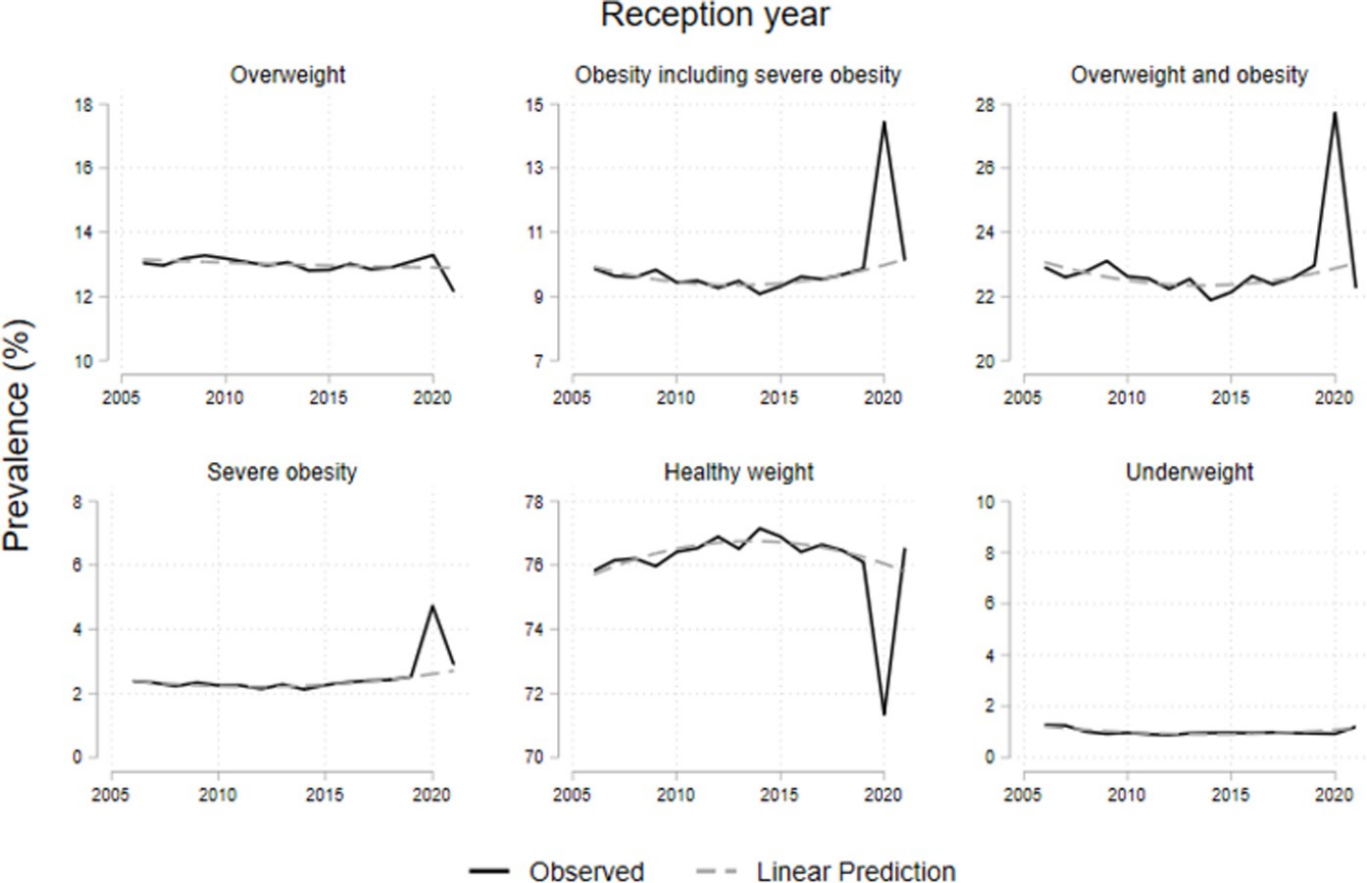
Prevalence evolution of children at reception year and year 6 by BMI. Note: Dotted lines represent the trends from 2006 to 2019. The category ‘Overweight and obesity’ includes severe obesity.

**Fig 2:**
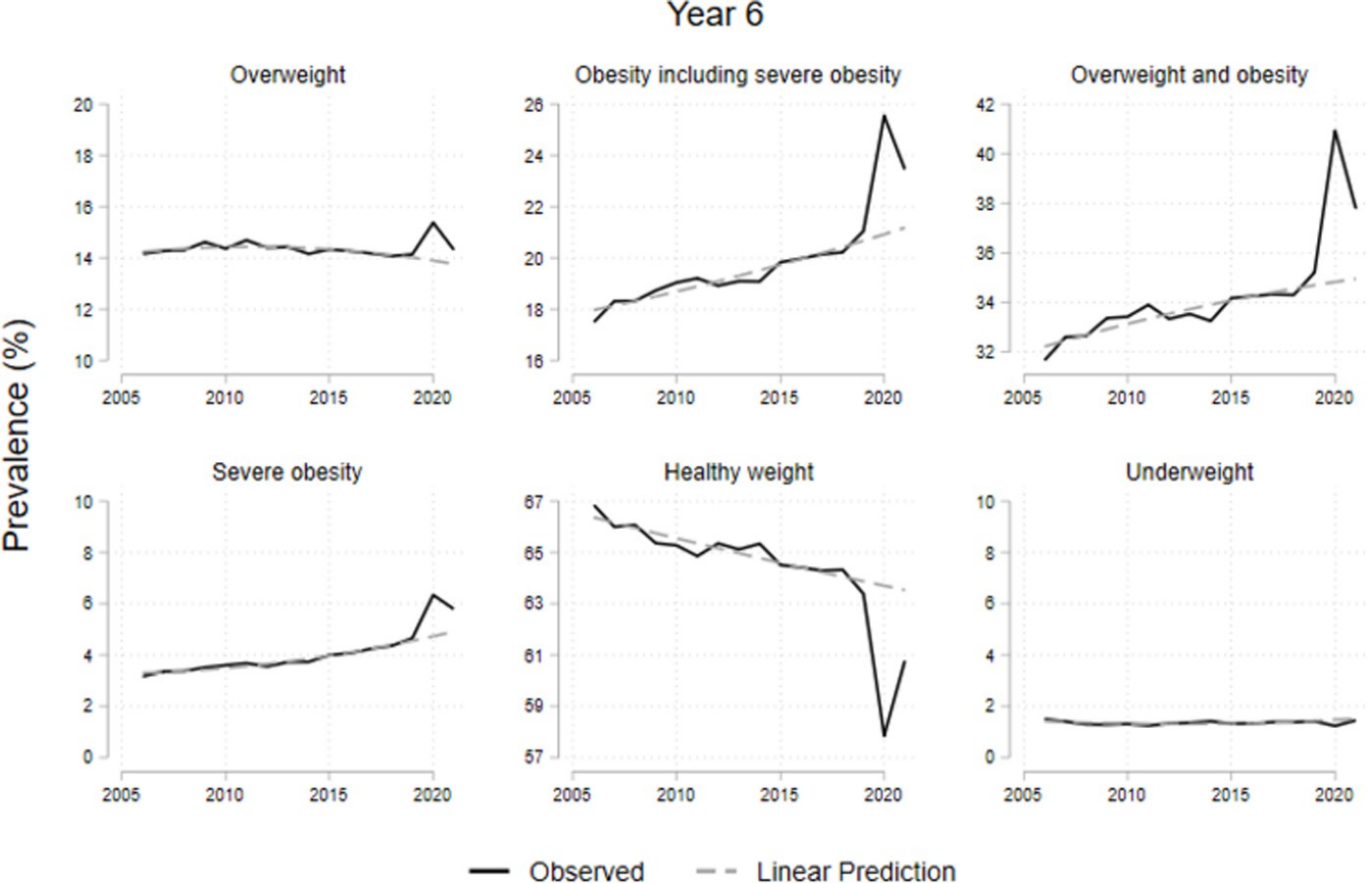
Prevalence evolution of children at year 6 by BMI. Note: Dotted lines represent the trends from 2006 to 2019. The category ‘Overweight and obesity’ includes severe obesity.

Table 1 shows the prevalence from 2006/07 to 2021/22 for children in reception (ages 4-5y) and year 6 (ages 10-11y). Both groups show an increase in overweight and obesity prevalence in 2020/21 and a decrease in 2021/22. Obesity prevalence among children aged 4-5y increased by 45%; from 9·9% in 2019/20 to 14·4% in 2020/21; and decreased to 10·1% in 2021/22, returning to the pre-pandemic trend (see Fig 1). For the same age-group, after an increase of 4·7 percentage points (pp) in 2020/21, the prevalence of overweight and obesity combined returned to pre-pandemic levels (22·3%) by 2021/22. For year 6 children, the prevalence of obesity and severe obesity, after a 4·5pp increase in 2020/21, showed a decrease of 2·1pp in 2021/22, remaining above the pre-pandemic previous trend (see Fig 2). We provide the data to replicate all our results (see S2 File).

**Table 1:** Historic observed percentage prevalence.

	2006/07	2010/11	2015/16	2019/20	2020/21	2021/22
	**Reception year**					
Underweight	1·3	1·0	1·0	0·9	0·9	1·2
(BMI ≥ 2^nd^ pc)	(1·2–1·3)	(0·9–1)	(0·9–1)	(0·9–1)	(0·9–1)	(1·2–1·2)
Healthy weight	75·8	76·4	76·9	76·1	71·3	76·5
(2^nd^ < BMI < 85^th^ pc)	(75·7–75·9)	(76·3–76·5)	(76·8–77)	(76–76·2)	(71·1–71·6)	(76·4–76·7)
Overweight	13·0	13·2	12·8	13·1	13·3	12·1
(85^th^ ≤ BMI < 95^th^ pc)	(12·9–13·1)	(13·1–13·3)	(12·7–12·9)	(13–13·2)	(13·1–13·5)	(12·1–12·2)
Obesity	7·5	7·2	7·0	7·3	9·7	7·2
(95^th^ ≤ BMI < 99·6^th^ pc)	(7·4–7·5)	(7·1–7·2)	(7–7·1)	(7·3–7·4)	(9·6–9·8)	(7·2–7·3)
Severe obesity	2·4	2·3	2·3	2·5	4·7	2·9
(BMI ≥ 99·6^th^ pc)	(2·3–2·4)	(2·2–2·3)	(2·2–2·3)	(2·5–2·6)	(4·6–4·8)	(2·8–2·9)
Obesity and severe obesity combined (BMI ≥ 95^th^ pc)	9·9	9·4	9·3	9·9	14·4	10·1
	(9·8–10)	(9·4–9·5)	(9·2–9·4)	(9·8–10)	(14·2–14·6)	(10–10·2)
Overweight, obesity and severe obesity combined (BMI ≥ 85^th^ pc)	22·9	22·6	22·1	23·0	27·7	22·3
	(22·8–23)	(22·5–22·7)	(22–22·2)	(22·8–23·1)	(27·5–28)	(22·1–22·4)
	**Year 6**					
Underweight	1·5	1·3	1·3	1·4	1·2	1·5
(BMI ≥ 2^nd^ pc)	(1·5–1·5)	(1·3–1·3)	(1·3–1·4)	(1·4–1·5)	(1·2–1·3)	(1·4–1·5)
Healthy weight	66·9	65·3	64·5	63·4	57·8	60·8
(2^nd^ < BMI < 85^th^ pc)	(66·7–67)	(65·1–65·4)	(64·4–64·6)	(63·3–63·5)	(57·6–58·1)	(60·7–60·9)
Overweight	14·2	14·4	14·3	14·1	15·4	14·3
(85^th^ ≤ BMI < 95^th^ pc)	(14·1–14·3)	(14·3–14·5)	(14·2–14·4)	(14–14·2)	(15·2–15·6)	(14·2–14·4)
Obesity	14·3	15·4	15·8	16·4	19·2	17·7
(95^th^ ≤ BMI < 99·6^th^ pc)	(14·3–15·3)	(15·4–15·5)	(15·8–15·9)	(16·3–16·4)	(19·1–19·3)	(17·6–17·7)
Severe obesity	3·2	3·6	4·0	4·7	6·3	5·8
(BMI ≥ 99·6^th^ pc)	(3·1–3·4)	(3·5–3·7)	(3·9–4)	(4·6–4·7)	(6·2–6·5)	(5·7–5·8)
Obesity and severe obesity combined (BMI ≥ 95^th^ pc)	17·5	19·0	19·8	21·0	25·5	23·4
	(17·4–18·8)	(18·9–19·1)	(19·7–19·9)	(20·9–21·2)	(25·3–25·8)	(23·3–23·6)
Overweight, obesity and severe obesity combined (BMI ≥ 85^th^ pc)	31·6	33·4	34·2	35·2	40·9	37·8
	(31·5–33·1)	(33·3–33·5)	(34–34·3)	(35·1–35·3)	(40·6–41·2)	(37·6–37·9)

BMI category prevalence in %

95% confidence intervals in parentheses.

Table 2 shows the regression results for each BMI category. The first row shows the change in prevalence for both age years associated to the years 2020/2021. Healthy weight prevalence dropped 4·3pp (p<·0001), obesity (including severe obesity) prevalence increased 3·5 pp (p<·0001) and the prevalence of severely obese increased 1·4 pp (p<·0001). The second row (Table 2) shows the change associated to the post-pandemic period (2020–2022). We can see that the changes in BMI category prevalence disappear (except for a marginal drop in overweight), implying a return to pre-pandemic trends for both ages combined. However, the post-pandemic effect on year 6 children (10-11y) persists as shown in the third row (Table 2). Healthy weight is 3·9 pp below previous trends (p<·0001); obesity prevalence is 3·2 pp (p<·0001) higher than the pre-pandemic period for 10-11-year-olds and overweight and obese combined is 4·0 pp (p<·0001) higher compared to previous levels.

**Table 2:** Changes in BMI category prevalence associated with the COVID-19 pandemic (percentage points).

	(1)	(2)	(3)	(4)	(5)	(6)
	Healthy weight (2^nd^<BMI <85^th^ pct)	Overweight (85^th^≤BMI<95^th^ pct)	Obesity (95^th^ ≤ BMI < 99·6^th^ pc)	Severe obesity (BMI≥99·6^th^ pc)	Obesity (including severe obesity) (BMI≥95^th^ pct)	Overweight and obesity (including severe obesity) (BMI≥85^th^ pct)
2020/2021	-4·3 [-5·9 - -2·7]	1·0 [0·9–1·1]	2·1 [1·4–2·8]	1·4 [0·5–2·3]	3·5 [1·9–5·0]	4·5 [2·8–6·1]
(Both year groups)	(p<·0001)	(p<·0001)	(p<·0001)	(0·005)	(p<·0001)	(p<·0001)
Year 6	-11·4 [-11·9 - -11·0]	1·3 [1·2–1·4]	8·2 [8·0–8·5]	1·5 [1·3–1·7]	9·7 [9·3–10·1]	11·1 [10·6–11·5]
	(p<·0001)	(p<·0001)	(p<·0001)	(p<·0001)	(p<·0001)	(p<·0001)
Post pandemic	1·0 [-0·3–2·3]	-0·5 [-0·8 - -0·2]	-0·4 [-1·2–0·3]	-0·1 [-0·7–0·5]	-0·5 [-1·8–0·8]	-1·0 [-2·3–0·3]
(Both year groups)	(0·140)	(0·002)	(0·227)	(0·797)	(0·422)	(0·112)
Year 6 x post pandemic	-3·9 [-4·8 - -3·0]	0·8 [0·7–1·0]	2·0 [1·6–2·4]	1·2 [0·7–1·6]	3·2 [2·3–4·1]	4·0 [3·1–4·9]
	(p<·0001)	(p<·0001)	(p<·0001)	(p<·0001)	(p<·0001)	(p<·0001)
Observations	32	32	32	32	32	32
R-squared	0·992	0·971	0·995	0·962	0·992	0·992
F-stat	916·6	4924	1879	145·8	787·8	902
Prob>F	0	0	0	0	0	0

Columns 1–6 indicate 6 different regression models with the dependent variables in the headings.

Observations refer to annual average by age group.

95% CI in brackets

P values in parenthesis

Figs 1 and 2 show observed trends in BMI category prevalence from 2006/07 to 2021/22 for children in reception and year 6 respectively, along with the expected values in the absence of the pandemic. These show a steep increase for both age groups in 2020/21. By 2021/22, reception year children but not year 6 children, return to the previous trends.

### Associations with social deprivation

**Fig 3:**
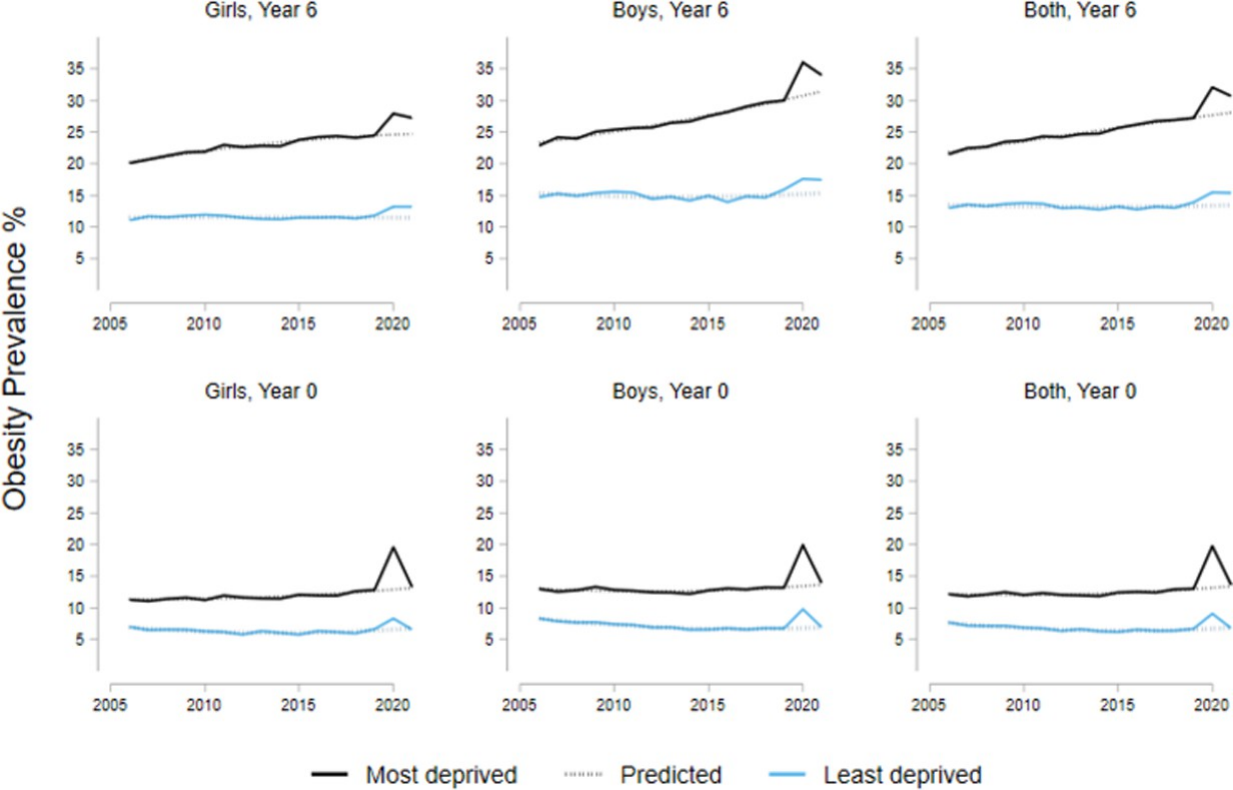
Obesity prevalence for children in the most and least deprived areas by sex. Note: Dotted lines represent the trends from 2006 to 2019. The category ‘Obese’ includes the severely obese.

Table 3 shows the changes in obesity prevalence during the pandemic for children in both age groups associated with level of deprivation. Among schools in the most deprived areas, the prevalence of childhood obesity was 8·5 pp (p<·0001) higher compared to the least deprived areas on average for the period 2006/21 (Table 3, first row). The increase in obesity prevalence associated with the COVID-19 pandemic (2020/2022) was 3·9 pp (p<·0001) greater in the most compared to the least deprived areas (Table 3). Year 6 children (10-11y) had on average a prevalence of obesity 9·4 pp (p<·0001) higher than reception year; and boys had an obesity prevalence 2·3 pp (p<·0001) higher than girls on average. Fig 3 illustrates the change in obesity prevalence for the least and most deprived areas for both age groups by sex.

**Table 3:** Changes in obesity prevalence associated with the COVID-19 pandemic for children in the most deprived areas (percentage points).

	(1)		(2)		(3)	
	Obese (including severely obese) (BMI≥95th pc)		Obese (including severely obese) (BMI≥95th pc)		Obese (including severely obese) (BMI≥95th pc)	
Most deprived vs least deprived	9·4	[8·4–10·5]	8·5	[7·7–9·2]	8·5	[7·7–9·2]
	(p<·0001)		(p<·0001)		(p<·0001)	
(both age groups)						
Year 6	10·8	[9·8–11·8]	9·6	[8·9–10·3]	9·4	[8·7–10·1]
	(p<·0001)		(p<·0001)		(p<·0001)	
Boys			2·4	[1·7–3·1]	2·3	[1·6–3·0]
(both age groups)			(p<·0001)		(p<·0001)	
2020/2021	2·6	[-0·7–5·8]	2·7	[-0·1–5·5]	2·7	[-0·0–5·4]
(both age groups)	(0·116)		(0·060)		(0·050)	
Post pandemic	-0·9	[-3·7–2·0]	-0·8	[-3·3–1·7]	-2·1	[-5·8–1·5]
(both age groups)	(0·546)		(0·526)		(0·244)	
Post pandemic * most deprived	3·6	[0·1–7·2]	3·9	[1·0–6·7]	3·9	[1·1–6·6]
	(0·045)		(0·008)		(0·006)	
(both age groups)						
Post pandemic * year 6					1·7	[-1·1–4·4]
					(0·233)	
Post pandemic * boys					1·0	[-1·7–3·8]
(both age groups)					(0·460)	
Observations	64		128		128	
R-squared	0·947		0·925		0·927	
F-stat	139·5		179·2		154·5	
Prob>F	0		0		0	

Columns 1–3 indicate 3 different regression models with the dependent variables in the headings

Observations in model (1) refer to annual averages by age and deprivation. Observations in models (2) and (3) also by sex.

95% CI in brackets

P values in parenthesis

### Variation by ethnicity

**Fig 4:**
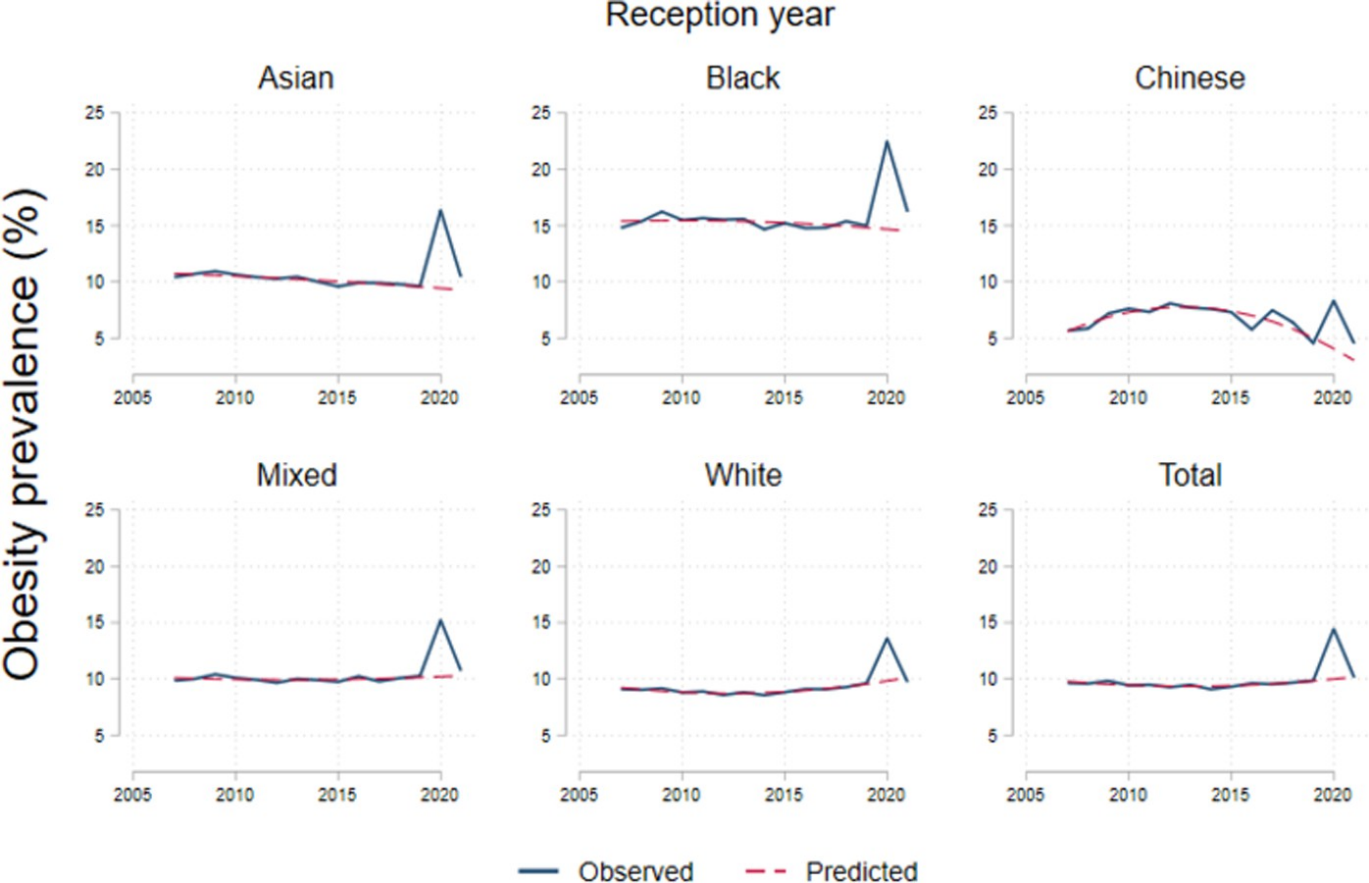
Obesity prevalence by ethnicity in reception year. Note: Dotted lines represent the trends from 2007 to 2019. The category ‘Obese’ includes the severely obese.

**Fig 5:**
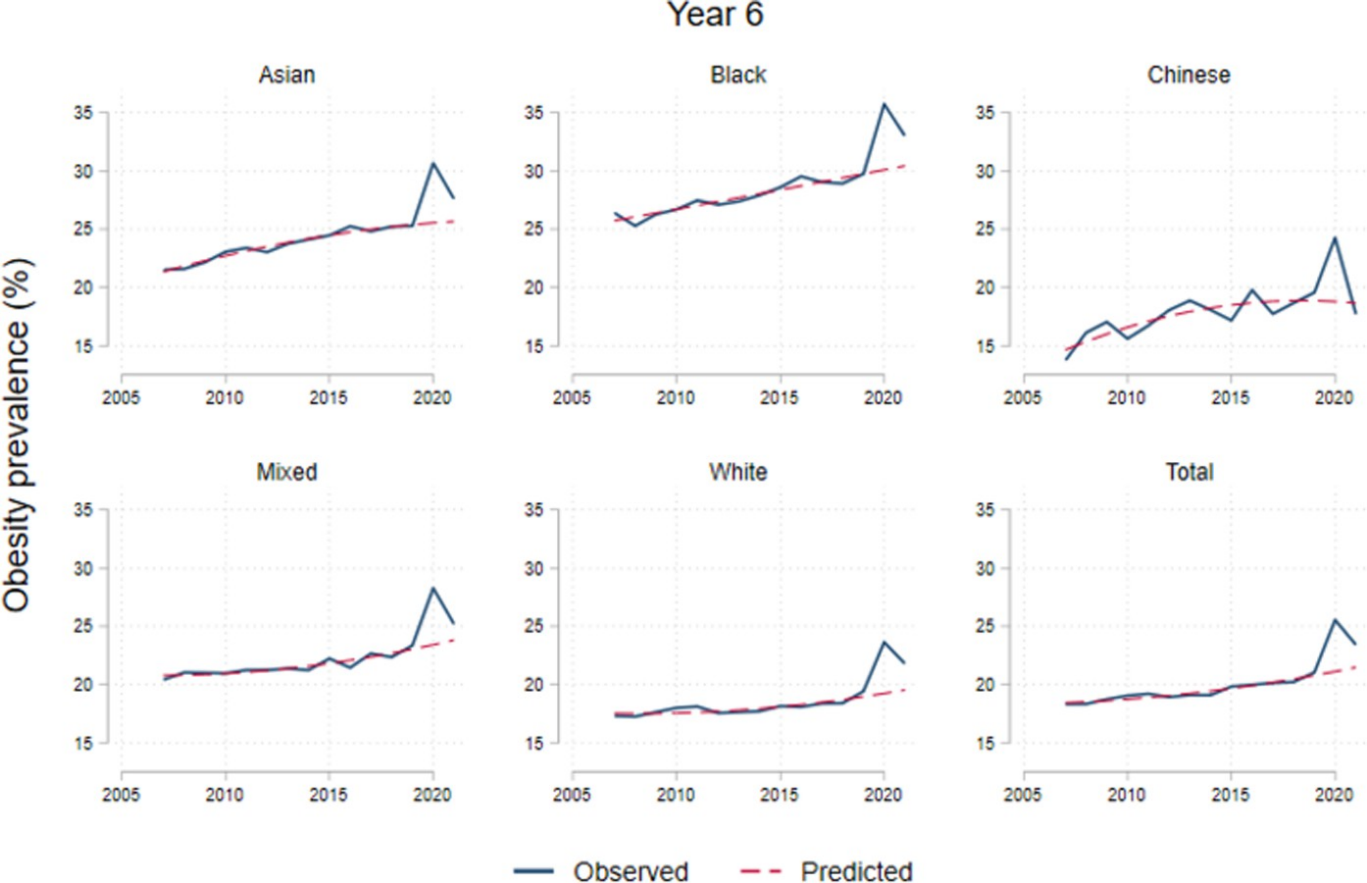
Obesity prevalence by ethnicity in year 6. Note: Dotted lines represent the trends from 2006 to 2019. The category ‘Obese’ includes severely obese.

Obesity prevalence trends during 2006/2022 by ethnicity are shown in Figs 4 and 5. Increases in obesity during 2020/2021 varied by ethnicity, among reception year children, Black children were the highest with 6.2pp (p < .001); followed by 5.6pp (p < .001) among South Asian; 4.2pp among Mixed (p < .001); 3.6pp (p < .001) for the White and 2.2pp (p = .06) among Chinese children. For 2021/2022, reception year children returned to previous trends. Table 4 shows differences in prevalence of obesity, severe obesity and both combined with overweight across ethnicities. Black ethnicity is associated with 7.1pp (p < .001) higher obesity prevalence than average, Asian 2.5pp (p < .001) higher. We did not find differences on the increase in BMI categories associated to the post pandemic period across ethnicities (Table 4).

**Table 4:** Changes in obesity prevalence associated to COVID-19 pandemic period by ethnicity (percentage points).

	(1)		(2)		(3)	
VARIABLES	Obese (including severely obese)		Severely obese		Overweight and obese (including severely obese)	
Asian	2.5	[1.6–3.3]	0.8	[0.7–1.0]	0.4	[-1.4–2.2]
	(p < .0001)		(p < .0001)		(0.663)	
Black	7.1	[6.4–7.8]	2.9	[2.2–3.6]	8.7	[7.9–9.4]
	(p < .0001)		(p < .0001)		(p < .0001)	
Chinese	-2.3	[-2.8 - -1.7]	-1.2	[-1.5 - -0.9]	-4.7	[-5.6 - -3.9]
	(p < .0001)		(p < .0001)		(p < .0001)	
Mixed	1.2	[0.8–1.7]	0.8	[0.4–1.2]	0.8	[0.2–1.4]
	(p < .0001)		(p < .0001)		(0.010)	
White	-1.0	[-1.2 - -0.7]	-0.4	[-0.6 - -0.3]	-0.8	[-1.2 - -0.4]
	(p < .0001)		(p < .0001)		(p < .0001)	
Other	3.3	[2.5–4.1]	1.0	[0.5–1.4]	3.6	[2.5–4.7]
	(p < .0001)		(p < .0001)		(p < .0001)	
Year 6	9.9	[9.7–10.1]	1.9	[1.8–2.1]	11.2	[10.8–11.6]
	(p < .0001)		(p < .0001)		(p < .0001)	
2020/2021	3.5	[2.4–4.6]	1.3	[0.7–1.9]	4.5	[3.1–5.9]
	(p < .0001)		(p < .0001)		(p < .0001)	
Post pandemic	-0.5	[-1.3–0.3]	0.1	[-0.3–0.5]	-1.0	[-2.2–0.2]
	(0.249)		(0.539)		(0.094)	
Asian*post pandemic	0.0	[-2.8–2.9]	0.1	[-0.4–0.5]	0.5	[-5.2–6.2]
	(0.974)		(0.780)		(0.862)	
Black*post pandemic	1.2	[-1.3–3.6]	0.6	[-0.8–1.9]	1.1	[-1.8–4.0]
	(0.347)		(0.414)		(0.453)	
Chinese*post pandemic	-3.1	[-4.0 - -2.2]	-0.9	[-1.6 - -0.2]	-2.6	[-4.5 - -0.7]
	(p < .0001)		(0.009)		(0.007)	
Mixed*post pandemic	-0.0	[-1.2–1.2]	-0.0	[-0.9–0.8]	-0.0	[-1.6–1.6]
	(0.980)		(0.914)		(0.956)	
White*post pandemic	-0.1	[-0.9–0.6]	-0.0	[-0.4–0.3]	-0.1	[-1.4–1.1]
	(0.760)		(0.896)		(0.835)	
Other*post pandemic	0.3	[-2.2–2.7]	0.5	[-0.5–1.5]	0.4	[-2.6–3.5]
	(0.830)		(0.301)		(0.783)	
Year 6 * 2020/2021	3.0	[2.3–3.7]	0.7	[0.4–1.0]	3.8	[2.7–4.9]
	(p < .0001)		(p < .0001)		(p < .0001)	
Observations	210		70		210	
R-squared	0.983		0.974		0.961	
F-stat	566.9		125.3		329.6	
Prob>F	0		0		0	

Columns 1–3 indicate 3 different regression models with the dependent variables in the headings

Observations refer to annual averages by ethnicity. The category severely obese is available from 2017/2018

95% CI in brackets

P values in parenthesis

### Projected lifelong BMI categories excess prevalence

Table 5 shows the estimated prevalence in the number of individuals for each BMI categories in excess of pre-pandemic levels. The first row shows the difference between observed and expected prevalence by BMI categories for 2021/22 for children of ages 10–11. The children of 4-5y are excluded from this part of the analysis as the age group returned to pre-pandemic levels. The following six rows indicate the projected evolution of those differences across time. Overweight prevalence increases rapidly and peaks around 25 years; obesity prevalence decreases in young age and starts increasing at around the age of 25 years, peaking at around 50 years; severe obesity decreases in young adulthood and starts increasing at around 35 years, peaking at 65 years. Overall, the number of overweight, obese, or severely obese population in the cohort increases until around 30 years and decreases after that. We provide the data to replicate all our results (see S2 File).

**Table 5:** Baseline and projected prevalence in excess of pre-pandemic levels.

	Overweight	Obesity	Severe obesity	Total
**Baseline**				
	85^th^ ≤ BMI < 95^th^	95^th^ ≤ BMI < 99^th^	BMI ≥ 99^th^	BMI ≥ 85^th^
**Age**				
10–11	11,168	27,919	16,752	55,838
	(9,772–13,960)	(22,335–33,503)	(9,772–22,335)	(41,879–69,798)
**Estimates**				
	BMI≥25, <30 kg/m2	BMI≥30, <40 kg/m2	BMI≥40	Total
**Age**				
16	25,021	25,607	16,721	67,349
	(28,935–31,389)	(19,073–26,673)	(10,234–15,747)	(58,243–73,808)
25	29,170	34,271	13,267	76,707
	(45,009–42,937)	(25,864–30,923)	(8,279–9,145)	(79,152–83,005)
35	16,903	39,654	14,801	71,358
	(37,737–33,397)	(34,141–36,819)	(10,373–9,491)	(82,251–79,706)
50	-11,405	40,472	19,997	49,065
	(1,910–5,942)	(42,609–42,911)	(15,624–15,799)	(64,176–60,620)
65	-27,828	36,670	20,239	29,082
	(-20,792 - -18,950)	(43,487–42,598)	(16,752–18,317)	(41,288–40,122)
75	-16,060	19,096	7,899	10,935
	(-14,134 - -13,879)	(22710–21833)	(7,190–7,629)	(16,020–15,328)

Notes: Data on 10–11 age groups was extrapolated to the total population in England from NCMP data.

Figures represent number of individuals.

With a population of 1.4 million 10-11y in England in 2020 [36], the 4pp (p < .001) increase in prevalence of overweight and obesity associated with the post-pandemic period, implies 55,838 (95% C.I. 42,275–68,402) additional children with an elevated BMI with respect to pre-pandemic trends. Of the additional children with an elevated BMI, 11,168 (95% C.I. 9,772–13, 960) were overweight; 27,919 (95% C.I. 22,335–33,503) were living with obesity, and 16,752 (95% C.I. 9,772–22,335) were living with severe obesity (see Table 5).

### Lifelong health costs

Table 6 shows per capita annual healthcare costs and total lifelong healthcare costs for the 55,838 children that became overweight, obese or severely obese during the COVID-19 pandemic. The total lifelong healthcare cost of the additional 4pp (95% CI 3.1–4.9) in overweight and obesity prevalence and 1 additional kg/m2 of in BMI is estimated to be £796 million (£788m – £804m) in 2021 values (see Table 6). The discounted value was £208 million (205–211) (Table 6). Coronary heart disease and type 2 diabetes made the largest contribution to lifelong healthcare costs, at about £700 million combined; cardiovascular disease, diabetes and osteoarthritis combined represent about 97% of the burden of disease in terms number of cases across time associated with elevated BMI (Table 6).

**Table 6:** Healthcare costs of main conditions and cost associated with losses in quality of life due to high BMI.

Condition	Annual per capita healthcare costs (2021 £)	Number of patients–years (thousands)	Total lifelong healthcare costs (£ millions)	Discounted lifelong healthcare costs (£ millions)	Utility lost (QALYs)	Utility lost costs (million £)	Discounted quality lost (QALYs)	Discounted utility lost cost (million £)
Coronary heart disease	2,269^1^	129.3 (128.5–129.9)	293.3 (291.5–294.8)	67 (66.5–67.4)	9,183 (9,139–9,219)	642.8 (639.7–645.3)	4,941 (4,904–4,972)	345.9 (343.3–348)
Stroke	994^1^	28.2 (28–28.4)	28 (27.8–28.2)	8.6 (8.5–8.7)	1,019 (1,010–1,026)	71.3 (70.7–71.8)	583 (576–588)	40.8 (40.3–41.2)
Type 2 diabetes	524^1^	777.2 (767.2–786)	407.3 (402–411.9)	105.8 (104–107.4)	55,184 (54,468–55,809)	3,862.9 (3,812.8–3,906.6)	30,225 (29,794–30,603)	2,115.7 (2,085.5–2,142.2)
Colorectal cancer	955^1^	0.8 (0.8–0.8)	0.8 (0.8–0.8)	0.2 (0.2–0.2)	31 (30–31)	2.1 (2.1–2.1)	17 (17–17)	1.2 (1.2–1.2)
Breast cancer	666^1^	0.3 (0.3–0.3)	0.2 (0.2–0.2)	0.05 (0.05–0.05)	6 (6–6)	0.4 (0.4–0.4)	3 (3–3)	0.2 (0.2–0.2)
Kidney cancer	729^1^	0.4 (0.4–0.4)	0.3 (0.3–0.3)	0.1 (0.1–0.1)	21 (21–21)	1.5 (1.5–1.5)	13 (13–13)	0.9 (0.9–0.9)
Endometrial cancer	2,915^2^	0.8 (0.8–0.8)	2.3 (2.3–2.4)	0.8 (0.8–0.8)	130 (128–132)	9.1 (9–9.2)	81 (79–82)	5.6 (5.6–5.7)
Oesophageal cancer	11,094^2^	0.6 (0.6–0.7)	7.2 (7.2–7.2)	2.3 (2.3–2.3)	21 (21–22)	1.5 (1.5–1.5)	13 (13–13)	0.9 (0.9–0.9)
Osteoarthritis	280^3^	203.8 (200.9–206.4)	57.1 (56.2–57.8)	23 (22.3–23.6)	20,741 (20,438–20,998)	1,451.9 (1,430.6–1,469.9)	13,638 (13,355–13,881)	954.7 (934.8–971.7)
**Total**		**1,141.5** **(1,127–1,154)**	**796.5** **(788.3–803.6)**	**207.9** **(204.7–210.7)**	**86,336** **(85,262–87,263)**	**6,043.5** **(5,968.3–6,108.4)**	**49,514** **(48,754–50,173)**	**3,466** **(3,412.8–3,512.1)**

Sources:

^1^Briggs et al (2018)

^2^ Adomako-Mensah et al (2020)

^3^ Brown et al. (2013)

### Losses in quality of life

The impact on quality of life is presented in Table 6. The loss in utility associated to additional 4pp (95% CI 3.1–4.9) increase in prevalence of overweight and obesity and 1 kg/m^2^ (0.9–1.09) of BMI was 86,336 (85,262–87,263) QALYs (49,514 (48,754–50,173) applying discount). The lifelong cost of this reduction in QALYs was £6 (5.9–6.1) billion (£3.5 (3.4–3.5) billion discounted). The condition with the highest utility losses was type 2 diabetes with a loss of 55,184 (54,468–55,809) QALYs (30,225 (29,794–30,603) applying discount). Type 2 diabetes incurred the highest cost of £3.9 (3.8–3.9) billion, about two-thirds of the total cost in quality of life of overweight and obesity, followed by osteoarthritis with £1.5 (1.4–1.5) billion and coronary heart disease at £642 (640–645) million.

### Productivity loss costs

The average number of annual days lost due to sickness per person was 3.3 (3.0–3.7) in healthy weight individuals, 3.9 (3.8–3.9) for the overweight population, and 5.4 (5.0–5.7) for the obese (including severely obese) population (S5 Table in S1 File) [33]. We estimate 3.8 (3.8–3.9) million days lost due to sickness absences overall with a cost of productivity lost associated with elevated BMI of £343 (338–346) million (Table 7). The income losses due to premature mortality for an average salary of £32,049 (S6 Table in S1 File) and a labour participation force of 76%, were £1.5 (1.5–1.5) billion. Overall, lifelong productivity losses were £1.9 (1.8–1.9) billion. The discounted value of productivity losses was £551 (547–555) million (Table 7).

**Table 7:** Costs of productivity loss.

	*Days / Years*	*Total cost (million £)*	*Discounted Costs (million £)*
Sickness absences (million days)	3.8 (3.8–3.9)	343 (338–346)	136 (134–139)
Premature mortality (000 years)	51.7 (51.6–51.7)	1,508 (1,505–1,514)	414 (413–416)
**Total cost**		**1,851 (1,843–1,860)**	**551 (547–555)**

### Total costs

Adding up healthcare costs, costs of reduced productivity and losses in quality-of-life costs, we obtained a lifelong cost of £8·7 (8·6–8·8) billion, which is associated to the increase of 4pp (95% C.I. 3·1–4·9) in overweight and obesity prevalence and an increase of 1 kg/m^2^ of BMI (0.9–1.09) (Table 8). We examine the sensitivity of our results to the increase in BMI levels varying that parameter by ·25 kg/m^2^. The total lifelong costs associated to a 4pp increase in overweight/obesity prevalence with a BMI increase of 0·75, and 1·25 kg/m^2^, amount to £6·7 and £13 billion, respectively. Those costs can be broken down in healthcare cost (£603m, £1·2b); productivity losses (£1·4b, £2·7b); and utility losses (£4·6b, £9·1b). We provide the data to replicate all our results (see S2 File).

**Table 8:** Total costs.

	Costs	Discounted
	(£ millions)	(£ millions)
Healthcare costs	796.5 (788.3–803.6)	207.9 (204.7–210.7)
Productivity losses	1,851 (1,843–1,860)	551 (547–555)
Losses in quality of life	6,043.5 (5,968.3–6,108.4)	3,466 (3,413–3,512)
**Total costs**	**8,691 (8,608–8,762)**	**4,225 (4,167–4,275)**

### Annual costs

Table 9 shows the evolution over time of the annual costs associated to the rise of 3·5pp (p<·0001) in childhood overweight and obesity prevalence and of 1 kg/m^2^ in BMI (0·75 and 1·25 in parentheses) during the COVID-19 pandemic. Yearly additional costs increase over time reaching a maximum around the age of 60 years. Similarly, costs related to the socioeconomic impact of reduced quality of life and income loss become higher through adulthood reaching a maximum during economically productive ages.

**Table 9:** Annual overweight and obesity related costs in excess of trends.

Year	Age	Annual healthcare costs (£ millions)	Annual cost to society excluding healthcare costs (£ millions)
2031	20	2·5 (2·4–2·8)	42·6 (39·5–46·7)
2041	30	2·9 (2·6–3)	44·6 (37·4–45)
2051	40	5·3 (4·4–5·2)	83·6 (68·9–82·8)
2061	50	14·7 (12–14·7)	171·7 (141·2–171·5)
2071	60	22·8 (18·1–23·2)	253·3 (204·4–255·7)
2081	70	17·9 (13·3–18·2)	114·6 (85·2–116·4)
2091	80	9·3 (5·6–8·7)	58·2 (35·8–54·6)

Annual costs associated to a 4pp increase in overweight and obesity and 1 kg/m^2^ in BMI.

In parentheses: Sensitivity analysis of annual costs associated to 4pp increase in overweight and obesity with a BMI increase of 0·75–1·25 kg/m^2^.

## Discussion

The projected healthcare and wider societal costs resulting from the increase in childhood overweight and obesity in England are substantial. The sharp increase in obesity prevalence during the COVID-19 pandemic illustrates the profound impacts of wider societal determinants. The persistence in older children after the pandemic is in keeping with other research that indicates that successful reversal in older age groups is highly problematic. In contrast, our finding of a rapid return to pre-pandemic levels in the youngest children suggests that a major shift in policy targeting under-fives is likely to be an effective means of tackling the growing population prevalence of overweight and obesity.

Our analysis indicates that the prevalence of overweight, obesity and severe obesity in England increased 4 percentage points (p<·0001) above expected values during the 2020–22 COVID-19 pandemic in the 10-11y old age group. This means that almost 56,000 additional children aged 10-11y became overweight or obese during the pandemic, and of these, 16,752 were severely obese. Our data raise profound social justice, equity, and financial concerns, with pressing implications for individuals, policymakers and UK society. Obesity prevalence in the most deprived areas of England is now more than double that in the least deprived and the gap has been widening over time.

In the absence of the pandemic, based on prior trends, the obesity prevalence observed in 2020/21 would not have occurred for at least another ten years. By 2021/22, overweight/obesity prevalence in children aged 4-5y had returned to the pre-pandemic level. However, it is possible that the cohort aged 4-5y in 2020/21 still has elevated BMI. Severe obesity prevalence in children aged 4-5y in 2021/22 remained slightly higher (0·2pp, p = ·002) with respect to historic trends.

The consequence of the rise in BMI and overweight/obesity prevalence among children aged 10-11y in England during the pandemic will be an additional burden of annual healthcare and societal costs reaching over £45 million in 2030 and incurring potentially 6 times higher annual costs over the future lifetime of the cohort. The lifelong cost to society amounts to £8·7 billion (£4·2 billion discounted). Our estimates of future costs should be regarded as a minimum as they do not include the impact of rising obesity prevalence in other age groups or the effects on future mental health.

Populations from more deprived areas are disproportionately affected given their higher prevalence of elevated BMI. Moreover, the higher increase in obesity prevalence among these populations, pose an excessive burden of lifelong economic costs compared to wealthier populations.

The rapid change in factors such as diet and physical activity, introduced by social restrictions to contain the virus, appears to have had varying impact across different populations. A systematic review and meta-analysis reported an increase of 2·67 kg and BMI of 0·94 kg/m^2^ in children during lockdowns [22]. However another meta-analysis of 36 studies, including 59,711 individuals over 16 years old from 32 countries, reported a global increase of 1·57 kg in adult body weight and 0·33 kg/m^2^ in BMI in the post-lockdown period May 2020 compared to pre-lock-down in March 2020 [37]. In our study the impact was greater among the younger cohort, but the return to previous prevalence levels was faster and 10-11y did not return to prior prevalence level after the pandemic.

The strengths of our study stem from the timely availability of national data on BMI in childhood, disaggregated by sex, deprivation status and ethnicity. Moreover, the availability of reliable information on healthcare cost of diseases in the UK, the risk of developing conditions, sickness days and mortality strengthen our results.

Our cost estimates are slightly higher compared to a meta-analysis of childhood obesity costs conducted before the pandemic [38]. Using the cost per child from that study and applying that to our population, the total cost would be £6.9b (6.2–8.1); the difference could be explained by the difference in methodologies. The previous paper does not consider losses in quality of life and use a different method to calculate productivity losses.

A limitation of this study is the repeated cross-sectional structure of the data. The cohort of 4–5-year-olds in 2020/2021 is different to the cohort of same age for the next year. Even though the obesity prevalence of children of reception year returned to the long-term trend, we did not observe the same children in 2021/2022 as they are in school year 1. It is possible that the obesity prevalence of that cohort in fact continued to increase. The same idea applies to the 10–11-year-old cohort. However, we believe that observing the cohort that comes behind provides valuable information to make a prediction. Independently of that, our results provide the lifelong excess costs of an increase in 4pp in the 10–11-year-old age group.

Information on deprivation levels is based on the school postal code, however, postal codes of residence may have different deprivation levels. Socioemotional impacts such as stigma and discrimination have an important effect on quality of life and mental health, but we were not able to include these in the cost estimates because relevant data were not available. Our estimates reflect associations, and we are unable to comment on causal determinants. Given available data, we are also unable to estimate relationships between overweight/obesity prevalence and individual factors such as lockdown, reduced face-to-face social interaction, changes in diet, and reduced physical activity.

The acute worsening of trends in obesity and overweight during the COVID-19 pandemic is however likely in large part to be an unintended consequence of measures to reduce transmission of the disease, alongside more direct effects of the pandemic itself on individuals and communities. Lockdowns led to the closure of businesses and education settings, economic hardship, social isolation, and an uncertain future, and had damaging effects on diets and physical activity [39].

Obesity, once established, has proven to be largely intractable. Hence, the potential long-term consequences for affected children are serious. Our lifelong estimates of elevated BMI prevalence are consistent with evidence showing that 60–85% of children with obesity remain obese in adulthood increasing their risks of future ill health [6]. People with obesity have higher risk of developing type 2 diabetes and BMI contributes to a large proportion of the risk of death and disability associated with diabetes [40]. There is hope in that children with obesity who are able to reduce their BMI to normal before adolescence have the same risk for developing type 2 diabetes as those who were never obese [10]. However, attempts to achieve sustained weight reduction have met with little success [10]. The implication is that prevention measures, to safeguard future generations of children against the scourge of overweight and obesity, must be a policy priority.

Our finding of a rapid return to pre-pandemic levels of overweight and obesity in the youngest children suggests that a major shift in policy targeting under-fives is likely to be an effective means of tackling the growing population prevalence of overweight and obesity. This is particularly pertinent as there are few preventive measures shown to be effective in adult populations even though this has long been the predominant policy focus. Our observation adds to the strong scientific evidence that health trajectories are established in early life (under age 5) and can then be amplified or attenuated through interventions and exposures that come into play at different points across the life-course. However, to effectively tackle childhood obesity, it is also important to reduce the risk of obesity during crucial life stages, which encompass the preconception and pregnancy periods, infancy and early childhood, as well as older childhood and adolescence [41].

Effective prevention therefore requires collaborative, co-ordinated, cross-sectoral interventions: current Department of Health and Social Care policies to combat obesity in England are widely thought to be insufficient [17]. The persistence of overweight and obesity in 10-11y olds and adults in our results highlight the importance of prevention at younger ages. However examples of interventions among adolescents and young people approaching parenthood include programmes to promote health literacy [42]. Such programmes have proven to be highly cost-effective, with incremental costs of about £120 [43], which is much lower compared to the cost of overweight of obesity, which we found to be between £650 and £4,400 per person per year. Preventive interventions that include collaborations between education and health departments based on a system-wide approach have also proved promising [17].

No country in the world has succeeded in curbing the rising population prevalence of overweight and obesity, even though the damaging consequences have been recognised for a long time. It took several decades of sustained advocacy to convince policymakers of the dangers of climate change and the necessity of reversing the decline in planetary health. The need to secure population health, starting with reversing the growing prevalence of overweight and obesity, also calls for sustained advocacy. Concerted action is possible but requires political will, a multisector approach, and acknowledgement that health is ultimately, a nation’s cardinal asset.

## Supporting information

S1 File Supporting information. (DOCX)

S2 File Underlying database. (XLSX)
