# Supplementary material for: Projected health and economic effects of the increase in childhood obesity during the COVID-19 pandemic in England: The potential cost of inaction
Source: PLoS One. 2024 Jan 24;19(1):e0296013. doi: 10.1371/journal.pone.0296013 (PMC10807834; doi:10.1371/journal.pone.0296013)
Supplement: S1 File — (DOCX) [file pone.0296013.s001.docx]

**Supporting information for:** **Projected health and economic effects of the increase in childhood obesity during the COVID-19 pandemic in England: the potential cost of inaction.**

I Ochoa-Moreno, R Taheem, K Woods-Townsend, D Chase, KM Godfrey, N Modi, M Hanson

**Appendix 2. Cost estimations inputs from the literature**

**Table S3. Risk of disease associated to BMI categories.**

| Disease | Age | HW | OW | OB | SO |
| --- | --- | --- | --- | --- | --- |
| Ischaemic disease | 20-64 | 1.1 | 1.5 | 2.9 | 5.6 |
|  | 65+ | 1.1 | 1.4 | 2.3 | 4.2 |
| Stroke | 20-64 | 1.0 | 1.2 | 1.6 | 2.2 |
|  | 65+ | 1.0 | 1.2 | 1.5 | 1.9 |
| Type 2 Diabetes | Adults | 1.0 | 2.4 | 6.1 | 12.4 |
| Colon cancer | 20-44 | 1.1 | 1.2 | 1.5 | 1.9 |
|  | 45+ | 1.0 | 1.1 | 1.4 | 1.8 |
| Breast cancer | 0-49 | 1.0 | 1.0 | 1.0 | 1.0 |
|  | 50+ | 1.0 | 1.1 | 1.3 | 1.3 |
| Kidney cancer | 20+ | 1.1 | 1.3 | 1.9 | 2.7 |
| Endometrial cancer | 20+ | 1.1 | 1.6 | 3.4 | 7.1 |
| Oesophageal cancer | 25+ | 1.1 | 1.6 | 3.0 | 6.0 |
| Osteoarthritis | Adults | 1.0 | 1.3 | 1.7 | 2.2 |

Source: Boyers et al. (2021)

| **Table S1. Healthcare costs per prevalent case per year** | | | |
| --- | --- | --- | --- |
| Condition | Cost inflated to 2021 | Source | year |
| Ischaemic disease | 2,269 | Briggs et al. (2018) | 2014 |
| Stroke | 994 | Briggs et al. (2018) | 2014 |
| Type 2 Diabetes | 524 | Briggs et al. (2018) | 2014 |
| Colon cancer | 955 | Briggs et al. (2018) | 2014 |
| Breast cancer | 666 | Briggs et al. (2018) | 2014 |
| Kidney cancer | 729 | Briggs et al. (2018) | 2014 |
| Endometrial cancer | 2,915 | Boyers et al. (2021) | 2016 |
| Oesophageal cancer | 11,094 | Boyers et al. (2021) | 2016 |
| Osteoarthritis | 138 | Brown et al. (2013) | 2012 |

| **Table S8. Inflation factors for England per year** | |
| --- | --- |
| 2011 | 1.19 |
| 2012 | 1.16 |
| 2013 | 1.13 |
| 2014 | 1.12 |
| 2015 | 1.12 |
| 2016 | 1.11 |
| 2017 | 1.08 |
| 2018 | 1.05 |
| 2019 | 1.03 |
| 2020 | 1.03 |
| 2021 | 1.00 |
| Source: Bank of England | |

| **Table S2. Utility lost associated to disease** | | | |
| --- | --- | --- | --- |
| **Condition** | **Prevalence** | **Incidence** | **Source** |
| Ischaemic disease | 0.070 | 0.070 | Briggs et al. (2019) |
| Stroke | 0.031 | 0.094 | Briggs et al. (2019) |
| Type 2 Diabetes | 0.071 |  | Briggs et al. (2019) |
| Colon cancer | 0.038 |  | Sullivan et al. (2011) |
| Breast cancer | 0.023 |  | Sullivan et al. (2011) |
| Kidney cancer | 0.048 |  | Briggs et al. (2019) |
| Endometrial cancer | 0.163 |  | Sullivan et al. (2011) |
| Oesophageal cancer | 0.033 |  | Sullivan et al. (2011) |
| Osteoarthritis | 0.102 |  | Sullivan et al. (2011) |

| **Table S5. Sickness absences per year by BMI category** | | | |
| --- | --- | --- | --- |
| Average* | Healthy weight | Overweight | Obesity |
| 4.1 | 3.3 (3.0 – 3.7) | 3.9 (3.8 – 3.9) | 5.4 (5.0 – 5.7) |

Sources: Estimations based on Virtanen et al. (2018)

* Office for National Statistics. Sickness absence in the UK labour market: 2020.

| **Table S6. Average salary in England, 2021** | |
| --- | --- |
| Annual | £32,049 |
| Daily | £89.03 |

Source: Office for National Statistics. Employment and Labour Market, 2021

| **Table S4. Morality rates and hazard ratios with 95% CI by age and BMI category** | | | | | | | | | | |
| --- | --- | --- | --- | --- | --- | --- | --- | --- | --- | --- |
| Age | Healthy weight | Overweight | | | Obesity | | | Severe Obesity | | |
|  | Mortality rate | HR | 95% CI | | HR | 95% CI | | HR | 95% CI | |
| 35 – 49 | 2.8% | 1.17 | 1.15 | 1.2 | 1.9 | 1.72 | 2.09 | 3.53 | 2.93 | 4.12 |
| 50 – 69 | 4.8% | 1.11 | 1.07 | 1.15 | 1.6 | 1.51 | 1.7 | 2.66 | 2.36 | 2.83 |
| 70 – 89 | 27.9% | 0.98 | 0.93 | 1.02 | 1.12 | 1.03 | 1.21 | 1.62 | 1.27 | 2.1 |

Source: Di Angelantonio et al. (2016)

| **Table S7. Discount rates** | | | |
| --- | --- | --- | --- |
|  | 1-30 years | 31-75 years | 75+ years |
| Costs | 3.5% | 3.0% | 2.5% |
| Utilities | 1.5% | 1.29% | 1.07% |

Source: The Green Book. Central Government guidance on appraisal and valuation. HM Treasury. 2022.

**References**

1. Briggs AD, Scarborough P, Wolstenholme J. Estimating comparable English healthcare costs for multiple diseases and unrelated future costs for use in health and public health economic modelling. PLoS One. 2018 May 24;13(5):e0197257.
2. Brown M, Marsh T, Rtveladze K, Fordham R, Suhrcke M, Turner D et al. Managing overweight and obesity among adults: report on economic modelling and cost consequence analysis. London: National Institute for Health and Care Excellence (NICE) , 2013. 83 p. (NICE).
3. Boyers, D., Retat, L., Jacobsen, E., Avenell, A., Aveyard, P., Corbould, E., Jaccard, A., Cooper, D., Robertson, C., Aceves-Martins, M. and Xu, B., 2021. Cost-effectiveness of bariatric surgery and non-surgical weight management programmes for adults with severe obesity: a decision analysis model. International Journal of Obesity, 45(10), pp.2179-2190.
4. Sullivan PW, Slejko JF, Sculpher MJ, Ghushchyan V. Catalogue of EQ-5D scores for the United Kingdom. Medical Decision Making. 2011 Nov;31(6):800-4.
5. The Green Book: appraisal and evaluation in central government. In: GOV.UK [Internet]. 18 Nov 2022 [cited 18 Oct 2023]. Available: <https://www.gov.uk/government/publications/the-green-book-appraisal-and-evaluation-in-central-governent/the-green-book-2020>
6. Virtanen M, Ervasti J, Head J, Oksanen T, Salo P, Pentti J, Kouvonen A, Väänänen A, Suominen S, Koskenvuo M, Vahtera J. Lifestyle factors and risk of sickness absence from work: a multicohort study. The Lancet Public Health. 2018 Nov 1;3(11):e545-54.
7. Office for National Statistics. Sickness absence in the UK labour market: 2020. Available at https://www.ons.gov.uk/employmentandlabourmarket/peopleinwork/labourproductivity/articles/sicknessabsenceinthelabourmarket/2020
8. Earnings and working hours - Office for National Statistics. 2021 [cited 18 Oct 2023]. Available: <https://www.ons.gov.uk/employmentandlabourmarket/peopleinwork/earningsandworkinghours/bulletins/annualsurveyofhoursandearnings/2021>
9. Di Angelantonio E, Bhupathiraju SN, Wormser D, Gao P, Kaptoge S, de Gonzalez AB, Cairns BJ, Huxley R, Jackson CL, Joshy G, Lewington S. Body-mass index and all-cause mortality: individual-participant-data meta-analysis of 239 prospective studies in four continents. The Lancet. 2016 Aug 20;388(10046):776-86.
10. Weir CB, Jan A. BMI Classification Percentile And Cut Off Points. PMID: 31082114.
